# C679X loss-of-function *PCSK9* variant is associated with lower fasting glucose in black South African adolescents: Birth to Twenty Plus Cohort

**DOI:** 10.1016/j.jcte.2019.100186

**Published:** 2019-02-28

**Authors:** Tinashe Chikowore, Venesa Sahibdeen, Liesl M. Hendry, Shane A. Norris, Julia H. Goedecke, Lisa K. Micklesfield, Zané Lombard

**Affiliations:** a Division of Human Genetics, School of Pathology, Faculty of Health Sciences, National Health Laboratory Service & University of the Witwatersrand, Johannesburg, South Africa; b DST-NRF Centre of Excellence in Mathematical and Statistical Sciences (CoE-MaSS), South Africa; c MRC/Wits Developmental Pathways for Health Research Unit, Department of Pediatrics, Faculty of Health Sciences, University of the Witwatersrand, Johannesburg, South Africa; d Sydney Brenner Molecular Institute of Biosciences, University of Witwatersrand, Johannesburg, South Africa; e Non-Communicable Diseases Research Unit, South African Medical Research Council, Cape Town, South Africa

**Keywords:** PCSK9, C679X, Fasting glucose, LDL-C, Type 1 diabetes

## Abstract

**Aim:**

To evaluate the association between loss-of-function (LOF) *PCSK9* variants (A433T/rs28362263 and C679X/rs28362286) and biomarkers of cardiometabolic risk, specifically fasting glucose and low density lipoprotein cholesterol (LDL-C) concentrations.

**Methods:**

Our study comprised 757 male and female black South African adolescents (mean age 18.0 ± 0.5 years) who are part of the Birth to Twenty Plus Cohort and had been genotyped for the two above-mentioned variants. Anthropometric measures were completed and fasting plasma glucose and lipid analysis were performed using standard procedures.

**Results:**

The median and interquartile range of fasting glucose and LDL-C for the whole group were 4.60 (4.36–4.88) mmol/L and 1.67 (1.25–2.14) mmol/L, respectively. After adjusting for sex, association between the biomarkers and A443T was not significant. However, C679X carriers displayed 0.30 [95% CI (−0.57, −0.02); p = 0.035] mmol/L lower fasting glucose and 0.50 [95% CI (−0.74, −0.26); p < 0.001) mmol/L lower LDL-C concentrations compared to non-carriers.

**Conclusions:**

Our results indicate for the first that the C679X variants associated with low fasting glucose levels during adolescents as had been known for LDL-C. In view that a similar finding was reported in older black South African adults, therefore, the correlation of lower fasting glucose and LDL-C levels with C679X is observed from an early age to adulthood.

## Introduction

Cardiovascular diseases (CVD) are the leading cause of mortality worldwide [1]. Elevated levels of low density lipoprotein cholesterol (LDL-C) are a major risk factor for CVDs [2]. Proprotein convertase subtilisin/kexin type 9 (PCSK9) is a key regulator of cholesterol homeostasis [3]. PCSK9 inhibitor drugs lower LDL-C levels by 60%, which exceeds traditional therapy with conventional statin drugs (30%) [2], [4]. PCSK9 is an enzyme that facilitates the degradation of the receptors for LDL-C (LDL-R) in the liver cells and it is encoded by the *PCSK9* gene [3]. LOF variants in this gene are associated with a reduction in the secretion or activity of PCSK9, thereby resulting in the increased availability of LDL-R which facilitates the removal of LDL-C from the blood and into the liver [3]. Conversely, gain-of-function variants for *PCSK9* result in high LDL-C levels and have been associated with familial hypercholesterolemia [2], [5]. Various studies among the young and old have shown that different LOF *PCSK9* variants are associated with low LDL-C concentrations in Caucasians and Africans due to allele frequency differences in these populations [5], [6], [7], [8]. Notably, low LDL-C concentrations have been associated with *PCSK9* variant R46L in Caucasians, and A443T, C679X, Y142X in people of African ancestry [5].

Although there are concerns that PCSK9 inhibitor drugs may be associated with increased T2D risk, similar to what has been demonstrated for statins [9], reports have been conflicting. A recent meta-analysis of PCSK9 inhibitor drug clinical trials indicated that these drugs had no effect on T2D risk [10]. However, a Mendelian randomization study presented evidence of causality between selected (LOF) *PCSK9* variants that are proxies for PCSK9 inhibitors, and increased risk of diabetes [4]. Conversely, lower PCSK9 levels associated with these drugs have been reported to be protective for T2D risk [11]. These discrepancies motivate the need for more studies to clarify the relationship between LOF *PCSK9* variants and glucose concentrations as biomarkers of diabetes.

Studies evaluating the association between LOF *PCSK9* variants and T2D risk have focused predominantly on R46L, which is common in Caucasians but very rare in people of African ancestry [9], [10], [12]. However, we reported recently that the C679X variant lowers fasting glucose levels in a longitudinal study among black South African adults, contrary to what has been reported for R46L in Caucasians [13]. These findings suggest that the LOF PCSK9 variants might have varied effects on T2D risk. It remains to be explored if the association between these LOF variants and fasting glucose observed in adults is similar during adolescence, a critical stage in the life course as it most often precedes the presentation of CVD risk factors. Therefore, this study aimed to investigate the association between A443T and C679X *PCSK9* LOF variants and fasting glucose and LDL-C concentrations in black South African adolescents.

## Materials and methods

### Study population

This study utilized data collected from the longitudinal Birth to Twenty (Bt20) Plus cohort of Soweto, South Africa, described in detail elsewhere [14]. Bt20 participants were enrolled at birth and detailed information has been collected from the participants at regular intervals. For this study, all participants with both DNA samples and phenotype data collected during the year-17 collection wave were included (n = 757).

Data and DNA sample collection from the Bt20 cohort received clearance from the Human Research Ethics Committee (Medical) of the University of the Witwatersrand (Wits HREC) under certificate number M010556. Likewise, this committee approved the use of DNA and data for this study under certificate number M120647. All participants provided written informed consent for the collection of data and samples, and subsequent analysis.

### Anthropometric measurements

Weight was measured with a digital scale (Dismed, SA), to the nearest 0.1 kg. Height was measured to the nearest 0.1 cm using a wall-mounted stadiometer (Holtain, UK), with individuals wearing light clothing and shoes removed. Body mass index (BMI) was computed as weight (measured in kilograms) divided by the square of the height (measured in meters) of an individual.

### Biochemical measurements

An auto-analyzer (Randox Daytona Clinical Analyzer Randox Laboratories, UK) that uses standard enzymatic methods was used to determine fasting plasma glucose, triglycerides, HDL-C and total cholesterol concentrations [15], [16]. LDL-C was calculated using the Friedwald equation. The coefficient of variation for these measures was less than 8% [17].

### Genotyping and quality control

DNA samples were extracted from whole blood using the salting-out method [18]. Genotyping was previously performed using the Illumina Metabochip (Illumina, USA) at the DNA Technologies Core of the University of California Davis (California, USA), and genotypes for the two single nucleotide polymorphisms (SNPs) of interest were extracted from data files. All details regarding the genotyping experiment and quality control of the genotypic data have been described previously [19].

### Statistical analysis

The normality of the continuous variables was evaluated using Q-Q plots (STATA 13 software package). Non-parametric tests were performed for the continuous descriptive characteristics as they were not normally distributed. The Mann-Whitney *U* test was used to determine whether continuous variables differed between males and females as indicated in Table 1. Spearman correlations were performed between the outcome variables (LDL-C and fasting glucose) and continuous variables (BMI and waist circumference). This information was used to identify covariates to include in the linear models for the associations between the biomarkers and selected *PCSK9* variants. The participants were of similar age (mean 18 ± 0.5 years), BMI was weakly correlated with fasting glucose (Spearman correlation = −0.03; p value = 0.442) and was not significantly different among the carriers and non-carriers of the LOF P*CSK9* variants (Table 2). Therefore age and BMI were not included as a covariates. The independent association of the C679X and A443T variants with fasting glucose and LDL-C was determined using generalized linear models, while adjusting for sex.

**Table 1:** Descriptive characteristics of the participants.

Variable	Male (n = 418)	Female (n = 339)	P value
Age (years)	17.9 (17.5–18.1)	17.9 (17.5–18.1)	0.532
BMI (kg/m^2^)	19.9 (18.7–21.7)	22.5 (20.1–25.7)	<0.001

*BMI category*			
Obese > 30 kg/m^2^ (n, %)	7 (1.7)	36 (10.6)	<0.001
Overweight 25–29.9 kg/m^2^ (n, %)	25 (6.0)	65 (19.2)	
Normal/underweight < 25 (n, %)	386 (92.3)	238 (70.2)	
Total cholesterol (mmol/L)	3.5 (3.0–4.0)	3.9 (3.3–4.5)	<0.001
Triglycerides (mmol/L)	0.7 (0.5–0.8)	0.6 (0.5–0.8)	0.239
HDL-C (mmol/L)	1.1 (0.9–1.2)	1.1 (0.9–1.36)	<0.001
LDL-C (mmol/L)	1.6 (1.1–2.0)	1.8 (1.4–2.3)	<0.001
Fasting glucose (mmol/L)	4.7 (4.4–5.0)	4.5 (4.3–4.8)	<0.001

Data presented as Median (IQR); BMI = body mass index; N = number; IQR = interquartile range.

**Table 2:** Characteristics of the carriers of the LOF *PCSK9* variants.

	C679X			A443T		
	Carriers (n = 34)	Non-Carriers (n = 723)	P value	Carriers (n = 72)	Non-Carriers (n = 684)	P value
BMI (kg/m^2^)	20.7 (18.9–23.8)	20.8 (19.1–23.4)	0.819	21.2 (19.8–24.3)	20.8 (19.0–23.4)	0.143
WC (cm)	71.8 (67.0–79.0)	72.0 (68.0–77.0)	0.826	73.5 (69.1–79.1)	72.0 (67.8–76.6)	0.091
Total Cholesterol (mmol/L)	3.1 (2.6–3.4)	3.7 (3.1–4.2)	<0.001	3.5 (3.1–4.4)	3.6 (3.1–4.2)	0.977
HDL-C (mmol/L)	1.0 (0.9–1.3)	1.1 (0.9–1.3)	0.504	1.1 (0.9–1.3)	1.1 (0.9–1.3)	0.918
Male/Female (n)	21/13	397/326	0.483	38/34	379/305	0.608

Data is presented as median(Interquartile range); kg/m^2^ = kilograms per meter squared; BMI = body mass index; WC = waist circumference

## Results

### Descriptive results

The descriptive statistics for the study cohort are shown in Table 1. There were significant differences in body mass index (BMI), fasting glucose, LDL-C, HDL-C and total cholesterol between the male and female participants. BMI, as well as lipids except triglycerides, were higher in women vs. men, but glucose was higher in men vs. women. Overall the participants were apparently health as indicated by the low mean LDL-C and fasting glucose levels, with only a few obese (5.7%) individuals.

The selected variants had a genotyping success rate of 99.95% and were in Hardy-Weinberg equilibrium. The minor allele frequencies for C679X and A443T were 0.02 and 0.05, respectively. There were no significant difference in the BMI and waist circumference of the carriers and non-carriers of the selected LOF *PCSK9* variants as illustrated in Table 2.

### Association between PCSK9 variants, LDL-C and fasting glucose

There were no significant associations between the selected *PCSK9* variants and BMI as illustrated in Table 2. BMI was not associated with fasting glucose levels (Pearson correlation −0.03; p value = 0.442) and therefore was not included as a covariate in the fasting glucose linear models. The heterozygous carriers of the C679X variants had significantly lower fasting glucose and LDL-C concentrations of 0.30 [95% CI (−0.57, − 0.02); p = 0.035] mmol/L and 0.50 [95% CI (−0.74, −0.26); p < 0.001) mmol/L compared to the non-carriers, respectively independent of sex (Table 3). There were no significant associations between A443T variants and LDL-C and fasting glucose.

**Table 3:** Associations of the selected *PCSK9* variants with fasting serum LDL-C and plasma glucose concentrations.

Variable	LDL-C				Fasting glucose			
	C679X		A443T		C679X		A443T	
	β (95%CI)	P value	β (95%CI)	P value	β (95%CI)	P value	β (95%CI)	P value
Carriersǂ	−0.50 (−0.74; − 0.26)	<0.001	−0.02 (−0.19; 0.15)	0.801	−0.30 (−0.57; − 0.02)	0.035	0.09 (−0.08; 0.27)	0.301
Sex*	0.21 (0.12; 0.31)	<0.001	0.22 (0.12; 0.32)	<0.001	−0.15 (−0.25; −0.04)	0.007	−0.15 (−0.26; 0.04)	0.006

ǂ Non-carriers is reference category.

* Male is the reference category; CI = confidence interval; kg/m^2^ = kilograms per meter squared; β = beta.

## Discussion

Our study set out to assess the association between LOF *PCSK9* variants (A443T and C679X) and LDL-C and fasting glucose concentrations in black South African adolescents We report for the first time that heterozygous carriers of the C679X significantly have lower fasting glucose levels than the non-carriers, after adjustment for sex in black South African adolescents. However, there were no significant associations in the carriers of A443T with LDL-C and fasting glucose.

The associations between the A443T and C679X variants and LDL-C have been documented extensively among African American adolescents [8], [20]. Similarly, in this study we have shown that the C679X variant was associated with a lower LDL-C concentration in black South African adolescents. In contrast, A443T variant was not associated with low LDL-C as has been reported in a study of black South African adults and in African American adolescents [13]. However, a study in African Canadians, also failed to show significant associations between A443T and LDL-C associations, thereby suggesting the possibility of other genetic and environmental factors that might influence the associations between A443T and LDL-C levels [7].

We observed significantly lower fasting glucose levels in heterozygous carriers of C679X compared to non-carriers independent of sex similarly to what we reported recently in black South African adults [13]. This study therefore, indicates that the C679X variant is associated with low fasting glucose from adolescents to adulthood in addition to the low LDL-C levels that had been documented before [5]. The selected *PCSK9* variants were not associated with BMI (Table 2), which was also not significantly associated with fasting glucose, thereby indicating that the low fasting glucose levels associated with C679X variant are not mediated by BMI, similar to what we reported in adult black South Africans[13]. Although the R46L variant has been reported to increase T2D risk in Caucasians, our findings further suggest that LOF *PCSK9* variants might have different effects with regards to T2D risk [13]. The lack of clarity of the precise mechanism which links the different *PCSK9* variants with biomarkers of T2D risk limits the interpretation of this discrepancy [21].

The C679X variant has a direct impact on protein function by limiting the exit of PCSK9 from the endoplasmic reticulum, leading to reduced levels of this enzyme [3]. Therefore its not surprising that the C679X variants is associated with low fasting glucose levels, since low PCSK9 levels have been reported to be protective in relation to T2D risk, while higher levels have been associated with an increased incidence of T2D and T1D in prospective cohort studies [11]. Furthermore, PCSK9 levels correlate positively with markers of glycemia such as fasting glucose and insulin resistance in adults and adolescents [11], [22]. On the other hand, the R46L variant leads to the substitution of the hydrophobic amino acid leucine with the hydrophilic arginine [3]. However, it is not clear how R46L affects PCSK9 function as it does not affect the levels of PCSK9 [3], [11].

Our study was limited in that we did not measure PCSK9 levels that would inform the relationship between the LOF *PCSK9* variants and fasting glucose. Ultimately, larger studies in people of African ancestry including oral glucose tolerance tests, which is the gold standard for T2D diagnosis, are required to validate these findings. Nonetheless, the association of the C679X variant with low fasting glucose in young participants that have no risk factors for diabetes, suggests that the PCSK9 inhibitor drugs which mimic these variants might even confer protection against both diabetes and hypercholesterolemia.

## Conclusion

In summary, our study reports for the first time that C679X LOF *PCSK9* variant is associated with lower fasting glucose levels in apparently healthy black South African adolescent cohort. In view of similar findings in adults, this variant is therefore associated with low fasting glucose and LDL-C from adolescents to adulthood. The association with lower fasting glucose concentrations is in contrast to what has been reported for other *PCSK9* variants in Caucasian populations. Therefore, it is plausible that different *PCSK9* LOF variants have varied effects on fasting glucose. More studies evaluating this phenomenon are required, and to determine which specific LOF *PCSK9* variants should be used as proxies to examine the effects of PCSK9 inhibitor drugs.
